# Juvenile xanthogranuloma as a new type of skin lesions in tuberous sclerosis complex

**DOI:** 10.1186/s13023-020-01396-7

**Published:** 2020-06-12

**Authors:** Qian Lu, Xiu-Yu Shi, Yang-Yang Wang, Meng-Na Zhang, Wen-Ze Wang, Jing Wang, Qiu-Hong Wang, Hui-Min Chen, Li-Ping Zou

**Affiliations:** 1 Department of Pediatrics, the First Medical Center of PLA General Hospital, Beijing, 100853 China; 2 Department of Pathology, Peking Union Medical College Hospital, Chinese Academy of Medical Sciences and Peking Union Medical College, Beijing, 100730 China; 3 Center for Brain Disorders Research, Capital Medical University, Beijing Institute for Brain Disorders, Beijing, 100069 China

**Keywords:** Tuberous sclerosis complex, Juvenile xanthogranuloma, Subependymal giant cell astrocytoma, Pathology, Sirolimus, Whole-exome sequencing

## Abstract

**Objective:**

Tuberous sclerosis complex (TSC) is a rare autosomal dominant genetic disease with many manifestations, and it involves any organ. In this study, we report a TSC patient with new type skin lesions.

**Methods:**

A 7-month-old TSC boy with multiple cutaneous nodules was admitted in our hospital. We collected the clinical data of the patient. We performed biopsy of cutaneous nodules and whole-exome sequencing in both paraffin block tissue and blood samples.

**Results:**

The patient presented with a 2 month history of gradual growth multiple cutaneous nodules. He had cardiac rhabdomyoma, subependymal giant cell astrocytoma (SEGA) and hypomelanotic macules. The pathological finding of cutaneous nodules was consistent with juvenile xanthogranuloma (JXG). After 3 months of sirolimus treatment, the multiple nodules disappeared. The whole-exome sequencing identified *TSC1* (c.2356C > T, p.R786*) mutation in both paraffin block tissue and blood samples. We overturned the original pathological diagnosis and finally identified JXG as a new type of skin lesions in TSC.

**Conclusion:**

This is the first report on the occurrence of JXG skin lesions in TSC patient. Genetic testing is necessary in JXG. These findings expand the phenotype of skin in patients with TSC and contribute to the elucidation of JXG pathogenesis and treatment.

## Background

TSC is a rare autosomal dominant genetic disease with an incidence rate of approximately 1/6000 [1]. TSC has many manifestations and involves any organ in the body. The most common findings are benign tumors of the skin, brain, heart, kidneys and lung. Skin lesions include hypomelanotic macules (90% of patients), facial angiofibromas (75% of patients), fibrous cephalic plaques (25% of patients) and shagreen patches (> 50% of patients) [1]. Some studies have reported the use of sirolimus on specific TSC manifestations [2–4].

JXG is a common form of non-Langerhans cell histiocytosis in infants and children. It is characterized by spontaneous formation of cutaneous nodules on the scalp, face, trunk and extremities. Approximately 71% of JXG cases occur during the first year of life [5]. The pathogenesis of JXG is unknown.

Here, we report a 7-month-old TSC boy who presented with a 2-month history of a gradual growth of multiple cutaneous nodules, which through biopsy and whole-exome sequencing finally identified JXG as a new type of skin lesions in TSC.

## Method

### Patients and diagnosis

A 7-month-old TSC boy with multiple cutaneous nodules was admitted in our hospital. We collected the clinical data of the patient. The diagnosis was made by at least two experienced specialists based on the 2012 TSC Consensus Conference updated diagnostic criteria [6]. We obtained written consent from his parents. This study was approved by the Ethics Committee of Chinese PLA General Hospital (Beijing, China).

We performed a biopsy of cutaneous nodules. Existing family members underwent testing all exons and introns of *TSC1* and *TSC2* genes. Paraffin block tissue and whole blood sample were collected from the patient to obtain genomic DNA for whole-exome sequencing.

We gave the patient sirolimos orally at the 8 months of age. The initial dose of sirolimus was 1 mg/(m^2^·day), and this dose was adjusted according to the blood concentration to maintain the blood concentration at 5–10 μg/L. He was administered oral sirolimus regularly for 1 year.

## Results

**Fig. 1:**
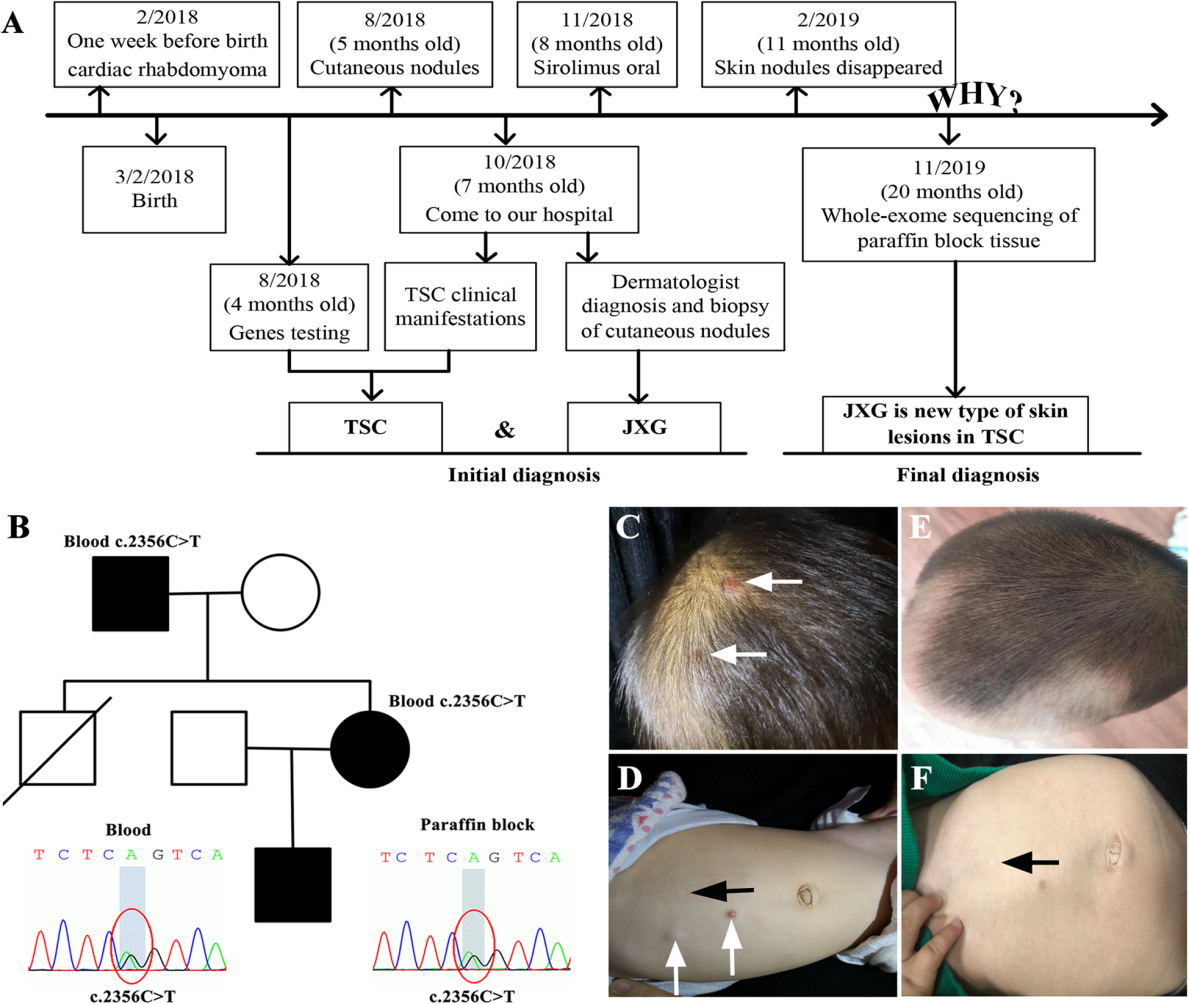
**a** The timeline of the patient. **b** Family pedigree. The black square indicates TSC patient. The square with slash indicates the person is died. Sequence chromatograms show *TSC1* mutation (c.2356C > T, p.R786*) in both paraffin block tissue and blood samples. The mutation is also found in blood of his mother and grandfather. **c**-**d** Nodules on the scalp and abdomen (white arrow) and hypomelanotic macule on the abdomen (black arrow) before treatment. **e**-**f** The multiple nodules disappeared and hypomelanotic macule in the abdomen was no change (black arrow) after 3 months of sirolimus

One week before birth, examination revealed a strong echo mass in the heart of the fetus (patient), indicating cardiac rhabdomyoma (Fig. 1a). A regular follow-up was recommended to observe changes in the cardiac rhabdomyoma. The patient’s mother had seizures since 4 years old. During pregnancy, she took lamotrigine and valpromide tablets but still had seizures every month. She had multiple skin lesions, including hypomelanotic macules, shagreen patches and facial angiofibromas. The patient’s uncle had seizures and died of “brain tumor” at 25 years old. The patient’s grandfather had hypomelanotic macules, shagreen patches and facial angiofibromas. When the patient was 4 months old, the mother was hospitalized. Existing family members had underwent testing for all exons and introns of *TSC1* and *TSC2* genes. Test results identified *TSC1* mutation (c.2356C > T, p.R786*) in the patient, his mother and grandfather (Fig. 1b). The nonsense mutation has been reported in a previous study [7]. Based on both clinical signs and genetic testing, our patient was definitely diagnosed with TSC.

**Fig. 2:**
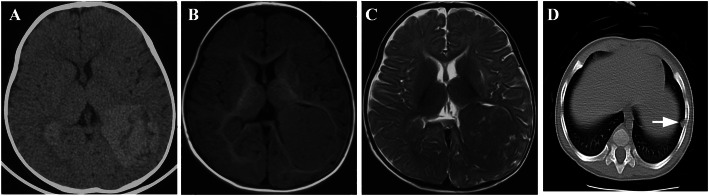
**a** Brain CT shows multiple punctate, flaky, and round-like high-density holes in the bilateral lateral ventricles and bilateral subependymal. A large lesion located in the posterior corner of the left lateral ventricle was diagnosed as SEGA. **b**-**c** Head MRI shows nodules in the posterior corners of bilateral ventricles on T1- and T2-weighted images. **d** Chest CT showed pulmonary subpleural nodule (white arrow)

The cutaneous nodules of the patient first appeared behind the right ear without evident cause or tenderness and gradually growth multiple nodules at 5-month old. After 2 months, he was admitted into our department. Physical examination showed multiple nodules on the scalp, eyelids, trunk and limbs of the patient which are papules or yellow and erythematous nodules without inflammation or ulceration (Fig. 1c-d). He had one hypomelanotic macule on the abdomen (Fig. 1d). Brain computed tomography (CT) showed multiple punctate, flaky and round-like high-density holes in the bilateral lateral ventricles and bilateral subependymal. A large lesion (51 mm × 46 mm) located in the posterior corner of the left lateral ventricle was diagnosed as subependymal giant cell astrocytoma (SEGA) (Fig. 2a). Head magnetic resonance imaging (MRI) showed nodules in the posterior corners of bilateral ventricles on T1- and T2-weighted images (Fig. 2b–c). Chest CT showed the presence of a pulmonary subpleural nodule in the lower left lobe (Fig. 2d). Cardiac ultrasound showed cardiac rhabdomyoma in the left ventricular cavity, which was approximately 15 mm × 12 mm. Abdominal ultrasonography was normal. Ophthalmologic evaluation was not performed, because the patient was too young to cooperatate. Laboratory blood tests showed that the patient had normal lipid level. He had no facial angiofibromas, shagreen patches or seizures, and his development was normal.

**Fig. 3:**
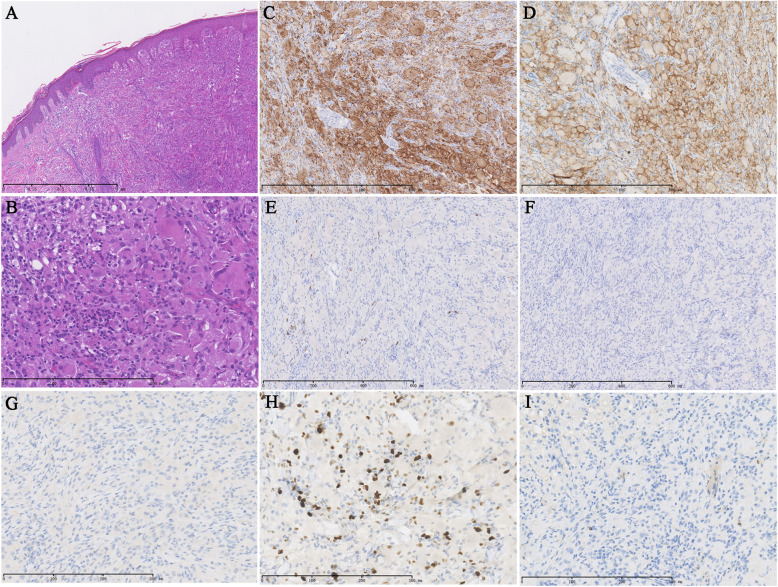
Histomorphologic examination. **a**-**b** Show typical histiocytosis findings in the dermis, with the presence of many multinucleated giant cells and inflammatory cells. **c**-**i** Show immunostainings results that are positive for CD68 (**c**) and CD168 (**d**). S-100 (**e**), CD1a (**f**), Lagerin (**g**) and Melan-A (**i**) are negative. The Ki-67 proliferation index is 15% (**h**)

The cutaneous nodules were different from common TSC skin lesions. We performed biopsy on the cutaneous nodules. Histopathological examination showed typical histiocytosis in the dermis with many multinucleated giant cells and inflammatory cells (Fig. 3a–b). Cluster differentiation 68 (CD68) and CD163 were positive (Fig. 3c–d) and S100, CD1a, langerin, human melanoma black 45 (HMB-45) and Melan-A were negative in immunohistochemical staining (Fig. 3e, f, g, and i). The Ki-67 (Fig. 3h) proliferation index was 15%. The pathological finding of cutaneous nodules was consistent with JXG. We believed that JXG and TSC simultaneously occurred in the patient.

We gave the patient sirolimus orally to treat TSC. After 3 months, the multiple nodules disappeared and hypomelanotic macule in the abdomen did not show any change (Fig. 1e-f). We were surprised that sirolimus had a significant effect on JXG. We performed whole-exome sequencing and identified *TSC1* mutation (c.2356C > T, p.R786*) in both paraffin block tissue and blood samples (Fig. 1b). No other disease-causing mutations were found. We considered JXG to be a new skin lesion of TSC.

Sirolimus was well tolerated without evident adverse reactions. After 1 year of administering sirolimus, no cutaneous nodule appeared. Cardiac ultrasound showed a reduction of cardiac rhabdomyoma in the left ventricular cavity (2 mm × 4 mm). Blood routine, liver functions, kidney functions and serum electrolytes were normal. The patient continued oral sirolimus and followed up regularly every 6 months.

## Discussion

Regarding to cutaneous nodules, a broad spectrum of differential diagnosis should be considered by immunohistochemical staining. S100, CD1a, Langerin are negative, thereby distinguishing the condition from Langerhans cell histiocytosis. S-100, HMB-45 and Melan-A are negative, thereby distinguishing the condition from malignant melanoma. The pathological finding is consistent with JXG.

Through whole-exome sequencing in paraffin block tissue, we overturned the original pathological diagnosis and finally identified JXG as a new type of skin lesions in TSC. The pathogenesis of JXG is unknown. Previously reported on patients with JXG have not performed whole-exome gene sequencing. Thus, no *TSC1* or *TSC2* mutations have been reported in JXG patients in previous studies.

The etiology of TSC involves the mutation in *TSC1* (9q34, encoding hamartin) or *TSC2* (16p13.3, encoding tuberin). Hamartin and tuberin form a functional unit that is involved in inhibiting the mammalian target of rapamycin (mTOR) pathway [8]. JXG patients have a prevalence of neurofibromatosis type 1 (NF1) that reached 18–30% [9]. Study reported that the mTOR pathway is hyperactivated in NF1 patients and mouse models [10]. We hypothesize that JXG may be related to mTOR pathway. Considering the finding in our case, we recommend that JXG patients should perform histopathological examination and genetic testing. Further studies are needed to find the relationship between JXG and the mTOR pathway.

Sirolimus selectively inhibits mTOR signaling. Clinical trials and scientific evidence support the use of sirolimus in TSC patients with specific manifestations, including SEGA and skin lesions [2–4]. Darcy AK et al. conducted a multicenter clinical investigation on the safety of mTOR inhibitors in TSC patients before the age of 2 years [11]. Sirolimus has been used to treat fetus with TSC and presenting cardiac rhabdomyoma [12]. Our patient started sirolimus treatment at the age of 8 months and the symptoms improved significantly, especially the JXG skin lesions.

The patient had pulmonary isolated subpleural nodule in the left lower lobe as observed via chest CT. The round mass in pulmonary nodules could be a sign of lung tumors or lesions. The lesion was not confirmed, because the parents of patient refused to perform lung biopsy. Annual CT test was recommended to the patient.

## Conclusion

This is the first report of JXG skin lesions in TSC patient. Genetic testing is necessary in JXG. The abovementioned findings expand the phenotype of skin in TSC and contribute to the elucidation of JXG pathogenesis and treatment.

## Data Availability

All the data in this study were included in the published article.

## Abbreviations

JXG: Juvenile xanthogranuloma
TSC: Tuberous sclerosis complex
SEGA: Sub-ependymal giant cell astrocytoma
CT: Computed tomography
MRI: Magnetic resonance imaging
CD68: Cluster differentiation 68
HMB45: Langerin, human melanoma black 45
mTOR: Mammalian target of rapamycin
